# 4-{3-[(2-Isopropyl-5-methyl­phen­oxy)meth­yl]-7*H*-1,2,4-triazolo[3,4-*b*][1,3,4]thia­diazin-6-yl}-3-(*p*-tol­yl)sydnone

**DOI:** 10.1107/S1600536810030205

**Published:** 2010-08-04

**Authors:** Jia Hao Goh, Hoong-Kun Fun, Nithinchandra, B. Kalluraya

**Affiliations:** a X-ray Crystallography Unit, School of Physics, Universiti Sains Malaysia, 11800 USM, Penang, Malaysia; b Department of Studies in Chemistry, Mangalore University, Mangalagangotri, Mangalore 574 199, India

## Abstract

In the title triazolothia­diazin compound, C_24_H_24_N_6_O_3_S (systematic name: 4-{3-[(2-isopropyl-5-methyl­phen­oxy)meth­yl]-7*H*-1,2,4-triazolo[3,4-*b*][1,3,4]thia­diazin-6-yl}-3-(4-methyl­phen­yl)-1,2,3-oxadiazol-3-ium-5-olate), an intra­molecular C—H⋯O hydrogen bond generates an *S*(6) ring motif. The two terminal methyl groups of the isopropyl unit are disordered over two sets of positions in a 0.715 (4):0.285 (4) ratio. The mean planes formed through the major and minor disordered isopropyl units are inclined at inter­planar angles of 73.1 (4) and 86.6 (8)°, respectively, with the attached phenyl ring. The 3,6-dihydro-1,3,4-thia­diazine ring adopts a twist-boat conformation. The inter­planar angle formed between 1,2,3-oxadiazole and 1,2,4-triazole rings is 18.80 (11)°. In the crystal, neighbouring mol­ecules are linked into sheets lying parallel to the *bc* plane by C—H⋯N hydrogen bonds. Weak inter­molecular π–π inter­actions [centroid–centroid distances = 3.2935 (11) and 3.5590 (12) Å] further stabilize the crystal structure.

## Related literature

For general background to and applications of materials related to the title triazolothia­diazine compound, see: Kalluraya & Rahiman (1997 ▶); Kalluraya *et al.* (2003 ▶); Newton & Ramsden (1982 ▶); Wagner & Hill (1974 ▶). For graph-set descriptions of hydrogen-bond ring motifs, see: Bernstein *et al.* (1995 ▶). For ring conformations and ring puckering analysis, see: Cremer & Pople (1975 ▶). For related structures, see: Goh *et al.* (2010**a* ▶,*b* ▶,*c* ▶,d* ▶). For the stability of the temperature controller used in the data collection, see: Cosier & Glazer (1986 ▶).
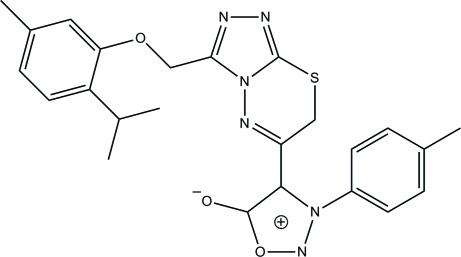


## Experimental

### 

#### Crystal data

C_24_H_24_N_6_O_3_S
*M* *_r_* = 476.55
Monoclinic, 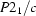

*a* = 16.7814 (3) Å
*b* = 7.2901 (1) Å
*c* = 20.2221 (3) Å
β = 106.991 (1)°
*V* = 2365.95 (6) Å^3^
*Z* = 4
Mo *K*α radiation
μ = 0.18 mm^−1^
*T* = 100 K
0.25 × 0.21 × 0.07 mm

#### Data collection

Bruker SMART APEXII CCD diffractometer
Absorption correction: multi-scan (*SADABS*; Bruker, 2009 ▶) *T*_min_ = 0.958, *T*_max_ = 0.988
20258 measured reflections
6897 independent reflections
4836 reflections with *I* > 2σ(*I*)
*R*_int_ = 0.053

#### Refinement

*R*[*F* ^2^ > 2σ(*F* ^2^)] = 0.057
*wR*(*F* ^2^) = 0.162
*S* = 1.04
6897 reflections
314 parameters
H-atom parameters constrained
Δρ_max_ = 0.68 e Å^−3^
Δρ_min_ = −0.60 e Å^−3^

### 

Data collection: *APEX2* (Bruker, 2009 ▶); cell refinement: *SAINT* (Bruker, 2009 ▶); data reduction: *SAINT*; program(s) used to solve structure: *SHELXTL* (Sheldrick, 2008 ▶); program(s) used to refine structure: *SHELXTL*; molecular graphics: *SHELXTL*; software used to prepare material for publication: *SHELXTL* and *PLATON* (Spek, 2009 ▶).

## Supplementary Material

Crystal structure: contains datablocks global, I. DOI: 10.1107/S1600536810030205/hb5569sup1.cif

Structure factors: contains datablocks I. DOI: 10.1107/S1600536810030205/hb5569Isup2.hkl

Additional supplementary materials:  crystallographic information; 3D view; checkCIF report

## Figures and Tables

**Table 1:** Hydrogen-bond geometry (Å, °)

*D*—H⋯*A*	*D*—H	H⋯*A*	*D*⋯*A*	*D*—H⋯*A*
C10—H10*B*⋯O3	0.97	2.20	2.993 (3)	138
C10—H10*A*⋯N1^i^	0.97	2.56	3.393 (3)	144
C15—H15*A*⋯N2^ii^	0.93	2.49	3.377 (3)	160
C19—H19*A*⋯N2^iii^	0.93	2.47	3.397 (3)	174

Symmetry codes: (i) 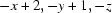; (ii) 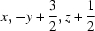; (iii) 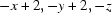.

**Fig. 1.**
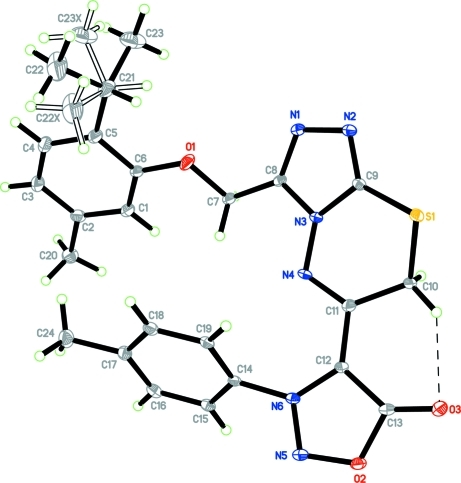
The molecular structure of (I), showing 30% probability displacement ellipsoids for non-H atoms. The minor disordered component is indicated as open bonds and an intramolecular hydrogen bond is shown as dashed line.

**Fig. 2.**
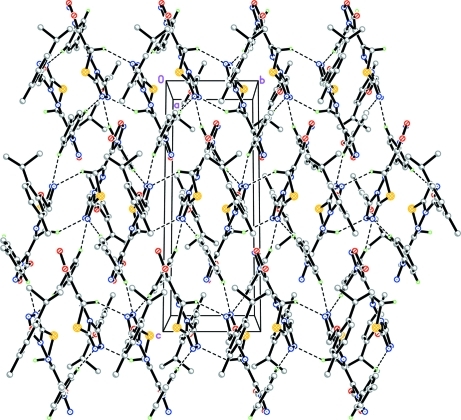
The crystal structure of (I), viewed along the a axis, showing a two-dimensional network parallel to the bc plane. Hydrogen atoms not involved in intermolecular interactions (dashed lines) have been omitted for clarity.

## supplementary crystallographic information

### Comment

Sydnones are a class of mesoionic compounds containing a 1,2,3-oxadiazole ring system. A number of sydnone derivatives have shown diverse biological activities such as anti-inflammatory, analgesic and anti-arthritic (Newton & Ramsden, 1982; Wagner & Hill, 1974) properties. Sydnones possessing heterocyclic moieties at the 4-position are also known for a wide range of biological properties (Kalluraya & Rahiman, 1997). Encouraged by these reports and in continuation of our research for biologically active nitrogen containing heterocycles, a triazolothiadiazine moiety at the 4-position of the phenylsydnone was introduced. A series of triazolothiadiazines were synthesized by the condensation of 4-bromoacetyl-3-arylsydnones with 3-aryloxymethyl-4-amino-5-mercapto-1,2,4-triazoles. 4-Bromoacetyl-3-arylsydnones were in turn obtained by the photochemical bromination of 4-acetyl-3-arylsydnones (Kalluraya *et al.*, 2003).

In the title triazolothiadiazine compound, an intramolecular C10—H10B···O3 hydrogen bond (Table 1) generates a six-membered ring, producing an *S*(6) hydrogen bond ring motif (Fig. 1, Bernstein *et al.*, 1995). The two terminal methyl groups of the isopropyl unit (atoms C22 and C23) were disordered over two positions with refined occupancies of 0.715 (4) and 0.285 (4). The mean planes formed through the major and minor disordered isopropyl units were inclined at interplanar angles of 73.1 (4) and 86.6 (8)°, respectively, with the attached C1-C6 phenyl ring. The 3,6-dihydro-1,3,4-thiadiazine ring (C9-C11/N3/N4/S1) adopts a twist-boat conformation. The puckering parameters are Q = 0.5952 (17) Å, θ = 113.75 (18)° and φ = 146.7 (2)° (Cremer & Pople, 1975). The essentially planar 1,2,3-oxadiazole (C12/C13/O2/N5/N6) and 1,2,4-triazole (C8/N1/N2/C9/N3) rings were inclined to each other at interplanar angle of 18.80 (11)°. The interplanar angles formed between the C1-C6 and C14-C19 phenyl rings with respect to 1,2,4-triazole and 1,2,3-oxadiazole rings are 49.56 (11) and 49.84 (11)°, respectively. The bond lengths and angles are comparable to those closely related structures (Goh *et al.*, 2010**a*,*b*,*c*,d*).

In the crystal structure, intermolecular C10—H10A···N1, C15—H15A···N2 and C19—H19A···N2 hydrogen bonds (Table 1) link neighbouring molecules into two-dimensional networks parallel to the *bc* plane (Fig. 2). Further stabilization of the crystal structure is provided by weak intermolecular *Cg*1···*Cg*1 [3.2935 (11) Å; symmetry code: -x+2, -y+1, -z] and *Cg*2···*Cg*3 [3.5590 (12) Å; symmetry code: -x+2, y-1/2, -z+1/2] interactions where *Cg*1, *Cg*2 and *Cg*3 are the centroids of 1,2,4-triazole, 1,2,3-oxadiazole and C14-C19 phenyl rings.

### Experimental

A solution of triazole (0.01 mol) and 4-bromoacetyl-3-tolylsydnone (0.01 mol) in absolute ethanol (20 ml) was heated under reflux for 10–12 h. The solution was concentrated, cooled to room temperature and neutrallized with 10 % sodium bicarbonate solution. The solid separated was filtered, washed with water, dried and recrystallized from ethanol. Yellow plates of (I) were obtained from a 1:2 mixture of DMF and ethanol by slow evaporation.

### Refinement

Atoms C22 and C23 are disordered over two sites with a refined occupancy ratio of 0.715 (4):0.285 (4). The same U^ij^ parameters were applied for atom pairs C6/C11, C22/C22X and C23/C23X. All hydrogen atoms were placed in their calculated positions, with C—H = 0.93–0.97 Å, and refined using a riding model, with *U*_iso_ = 1.2 or 1.5 *U*_eq_(C). The rotating group model was used for the methyl groups.

### Crystal data

**Table:**

C_24_H_24_N_6_O_3_S	*F*(000) = 1000
*M**_r_* = 476.55	*D*_x_ = 1.338 Mg m^−^^3^
Monoclinic, *P*2_1_/*c*	Mo *K*α radiation, λ = 0.71073 Å
Hall symbol: -P 2ybc	Cell parameters from 3586 reflections
*a* = 16.7814 (3) Å	θ = 3.7–30.0°
*b* = 7.2901 (1) Å	µ = 0.18 mm^−^^1^
*c* = 20.2221 (3) Å	*T* = 100 K
β = 106.991 (1)°	Plate, yellow
*V* = 2365.95 (6) Å^3^	0.25 × 0.21 × 0.07 mm
*Z* = 4	

### Data collection

**Table:**

Bruker SMART APEXII CCD diffractometer	6897 independent reflections
Radiation source: fine-focus sealed tube	4836 reflections with *I* > 2σ(*I*)
graphite	*R*_int_ = 0.053
φ and ω scans	θ_max_ = 30.2°, θ_min_ = 3.5°
Absorption correction: multi-scan (*SADABS*; Bruker, 2009)	*h* = −23→23
*T*_min_ = 0.958, *T*_max_ = 0.988	*k* = −10→9
20258 measured reflections	*l* = −28→26

### Refinement

**Table:**

Refinement on *F*^2^	Primary atom site location: structure-invariant direct methods
Least-squares matrix: full	Secondary atom site location: difference Fourier map
*R*[*F*^2^ > 2σ(*F*^2^)] = 0.057	Hydrogen site location: inferred from neighbouring sites
*wR*(*F*^2^) = 0.162	H-atom parameters constrained
*S* = 1.04	*w* = 1/[σ^2^(*F*_o_^2^) + (0.0787*P*)^2^ + 0.8879*P*] where *P* = (*F*_o_^2^ + 2*F*_c_^2^)/3
6897 reflections	(Δ/σ)_max_ = 0.002
314 parameters	Δρ_max_ = 0.68 e Å^−^^3^
0 restraints	Δρ_min_ = −0.60 e Å^−^^3^

### Special details

**Table:**

Experimental. The crystal was placed in the cold stream of an Oxford Cryosystems Cobra open-flow nitrogen cryostat (Cosier & Glazer, 1986) operating at 100.0 (1)K.
Geometry. All esds (except the esd in the dihedral angle between two l.s. planes) are estimated using the full covariance matrix. The cell esds are taken into account individually in the estimation of esds in distances, angles and torsion angles; correlations between esds in cell parameters are only used when they are defined by crystal symmetry. An approximate (isotropic) treatment of cell esds is used for estimating esds involving l.s. planes.
Refinement. Refinement of F^2^ against ALL reflections. The weighted R-factor wR and goodness of fit S are based on F^2^, conventional R-factors R are based on F, with F set to zero for negative F^2^. The threshold expression of F^2^ > 2sigma(F^2^) is used only for calculating R-factors(gt) etc. and is not relevant to the choice of reflections for refinement. R-factors based on F^2^ are statistically about twice as large as those based on F, and R- factors based on ALL data will be even larger.

### Fractional atomic coordinates and isotropic or equivalent isotropic displacement parameters (Å^2^)

**Table:**

	*x*	*y*	*z*	*U*_iso_*/*U*_eq_	Occ. (<1)
S1	1.12872 (3)	0.84837 (7)	0.02404 (3)	0.01713 (12)	
O1	0.76141 (9)	0.7367 (3)	−0.02658 (8)	0.0334 (4)	
O2	1.15782 (9)	1.0448 (2)	0.31193 (7)	0.0200 (3)	
O3	1.24221 (9)	0.9624 (2)	0.24693 (8)	0.0237 (3)	
N1	0.90823 (10)	0.6471 (2)	−0.06230 (9)	0.0171 (3)	
N2	0.98873 (10)	0.6914 (2)	−0.06497 (9)	0.0174 (3)	
N3	0.97931 (9)	0.7563 (2)	0.03891 (8)	0.0135 (3)	
N4	0.99250 (10)	0.8392 (2)	0.10282 (8)	0.0146 (3)	
N5	1.07442 (10)	1.0591 (2)	0.30466 (9)	0.0185 (3)	
N6	1.03703 (10)	1.0015 (2)	0.24167 (8)	0.0150 (3)	
C1	0.67088 (12)	0.6673 (3)	0.04511 (11)	0.0240 (4)	
H1A	0.7144	0.6128	0.0788	0.029*	
C2	0.59151 (13)	0.6753 (3)	0.05414 (11)	0.0232 (4)	
C3	0.52834 (13)	0.7576 (3)	0.00321 (12)	0.0285 (5)	
H3A	0.4750	0.7640	0.0081	0.034*	
C4	0.54356 (13)	0.8308 (4)	−0.05515 (13)	0.0298 (5)	
H4A	0.4999	0.8863	−0.0883	0.036*	
C5	0.62153 (12)	0.8244 (3)	−0.06609 (11)	0.0242 (5)	
C6	0.68510 (12)	0.7400 (3)	−0.01377 (10)	0.0181 (3)	
C7	0.83038 (11)	0.6680 (3)	0.02616 (10)	0.0178 (4)	
H7A	0.8219	0.5400	0.0354	0.021*	
H7B	0.8383	0.7374	0.0685	0.021*	
C8	0.90403 (11)	0.6888 (3)	−0.00054 (10)	0.0148 (4)	
C9	1.02919 (11)	0.7570 (3)	−0.00414 (10)	0.0149 (4)	
C10	1.14197 (11)	0.7915 (3)	0.11406 (10)	0.0164 (4)	
H10A	1.1460	0.6594	0.1199	0.020*	
H10B	1.1934	0.8451	0.1426	0.020*	
C11	1.06984 (12)	0.8615 (3)	0.13761 (10)	0.0181 (3)	
C12	1.09001 (11)	0.9487 (3)	0.20486 (10)	0.0150 (4)	
C13	1.17215 (12)	0.9794 (3)	0.25034 (10)	0.0178 (4)	
C14	0.94665 (11)	1.0107 (3)	0.22300 (10)	0.0148 (4)	
C15	0.90726 (12)	0.9404 (3)	0.26880 (11)	0.0181 (4)	
H15A	0.9378	0.8843	0.3097	0.022*	
C16	0.82111 (13)	0.9555 (3)	0.25236 (11)	0.0212 (4)	
H16A	0.7937	0.9074	0.2824	0.025*	
C17	0.77504 (12)	1.0416 (3)	0.19151 (12)	0.0220 (4)	
C18	0.81788 (13)	1.1146 (3)	0.14753 (11)	0.0213 (4)	
H18A	0.7880	1.1733	0.1071	0.026*	
C19	0.90384 (12)	1.1014 (3)	0.16288 (10)	0.0173 (4)	
H19A	0.9318	1.1518	0.1337	0.021*	
C20	0.57629 (14)	0.5967 (4)	0.11855 (13)	0.0304 (5)	
H20A	0.5324	0.5076	0.1057	0.046*	
H20B	0.5606	0.6935	0.1444	0.046*	
H20C	0.6263	0.5392	0.1464	0.046*	
C21	0.63844 (15)	0.8953 (4)	−0.13113 (13)	0.0365 (6)	
H21A	0.6957	0.9417	−0.1180	0.044*	0.715 (4)
H21B	0.6927	0.8569	−0.1322	0.044*	0.285 (4)
C22	0.5837 (4)	1.0436 (9)	−0.1679 (3)	0.0728 (19)	0.715 (4)
H22A	0.5845	1.1429	−0.1365	0.109*	0.715 (4)
H22B	0.5278	0.9981	−0.1858	0.109*	0.715 (4)
H22C	0.6030	1.0865	−0.2054	0.109*	0.715 (4)
C23	0.6338 (3)	0.7265 (7)	−0.1815 (2)	0.0531 (11)	0.715 (4)
H23A	0.6492	0.7659	−0.2214	0.080*	0.715 (4)
H23B	0.5780	0.6789	−0.1958	0.080*	0.715 (4)
H23C	0.6715	0.6324	−0.1579	0.080*	0.715 (4)
C22X	0.6361 (9)	1.129 (2)	−0.1228 (8)	0.0728 (19)	0.285 (4)
H22D	0.6563	1.1860	−0.1576	0.109*	0.285 (4)
H22E	0.6709	1.1645	−0.0779	0.109*	0.285 (4)
H22F	0.5800	1.1684	−0.1283	0.109*	0.285 (4)
C23X	0.5773 (8)	0.8472 (19)	−0.1949 (5)	0.0531 (11)	0.285 (4)
H23D	0.5744	0.7161	−0.1995	0.080*	0.285 (4)
H23E	0.5928	0.8992	−0.2330	0.080*	0.285 (4)
H23F	0.5239	0.8940	−0.1948	0.080*	0.285 (4)
C24	0.68205 (13)	1.0618 (4)	0.17411 (14)	0.0315 (5)	
H24A	0.6614	0.9817	0.2030	0.047*	
H24B	0.6569	1.0302	0.1265	0.047*	
H24C	0.6685	1.1864	0.1817	0.047*	

### Atomic displacement parameters (Å^2^)

**Table:**

	*U*^11^	*U*^22^	*U*^33^	*U*^12^	*U*^13^	*U*^23^
S1	0.0194 (2)	0.0186 (3)	0.0151 (2)	−0.00106 (17)	0.00760 (17)	0.00018 (19)
O1	0.0171 (7)	0.0642 (13)	0.0177 (8)	0.0052 (7)	0.0031 (6)	0.0140 (8)
O2	0.0215 (7)	0.0234 (8)	0.0134 (7)	−0.0021 (5)	0.0025 (5)	−0.0027 (6)
O3	0.0196 (7)	0.0290 (9)	0.0212 (8)	−0.0027 (6)	0.0037 (6)	−0.0027 (7)
N1	0.0191 (7)	0.0171 (9)	0.0135 (8)	−0.0002 (6)	0.0024 (6)	−0.0001 (7)
N2	0.0214 (7)	0.0175 (9)	0.0130 (8)	0.0004 (6)	0.0044 (6)	−0.0014 (6)
N3	0.0178 (7)	0.0124 (8)	0.0106 (7)	0.0003 (6)	0.0046 (6)	−0.0008 (6)
N4	0.0208 (7)	0.0140 (8)	0.0098 (7)	0.0000 (6)	0.0058 (6)	−0.0023 (6)
N5	0.0224 (8)	0.0182 (9)	0.0135 (8)	−0.0013 (6)	0.0031 (6)	−0.0026 (7)
N6	0.0200 (7)	0.0139 (8)	0.0108 (7)	−0.0002 (6)	0.0043 (6)	−0.0001 (6)
C1	0.0202 (9)	0.0315 (12)	0.0183 (10)	0.0003 (8)	0.0025 (8)	0.0001 (9)
C2	0.0239 (9)	0.0267 (12)	0.0195 (10)	−0.0044 (8)	0.0070 (8)	−0.0046 (9)
C3	0.0193 (9)	0.0385 (14)	0.0282 (12)	0.0028 (9)	0.0077 (8)	0.0007 (11)
C4	0.0204 (9)	0.0417 (15)	0.0261 (12)	0.0075 (9)	0.0053 (8)	0.0043 (11)
C5	0.0211 (9)	0.0322 (13)	0.0180 (10)	0.0014 (8)	0.0036 (8)	0.0028 (9)
C6	0.0178 (6)	0.0223 (8)	0.0137 (6)	−0.0003 (5)	0.0038 (5)	0.0003 (6)
C7	0.0171 (8)	0.0220 (11)	0.0126 (9)	0.0000 (7)	0.0018 (7)	0.0009 (8)
C8	0.0168 (8)	0.0131 (9)	0.0122 (9)	0.0004 (6)	0.0003 (7)	0.0017 (7)
C9	0.0188 (8)	0.0133 (9)	0.0132 (9)	0.0018 (7)	0.0057 (7)	0.0016 (7)
C10	0.0172 (8)	0.0193 (10)	0.0117 (9)	0.0014 (7)	0.0025 (7)	−0.0011 (7)
C11	0.0178 (6)	0.0223 (8)	0.0137 (6)	−0.0003 (5)	0.0038 (5)	0.0003 (6)
C12	0.0170 (8)	0.0161 (10)	0.0110 (8)	−0.0007 (7)	0.0028 (7)	0.0005 (7)
C13	0.0231 (9)	0.0168 (10)	0.0123 (9)	−0.0020 (7)	0.0033 (7)	−0.0007 (8)
C14	0.0170 (8)	0.0151 (9)	0.0112 (9)	0.0011 (7)	0.0026 (7)	−0.0024 (7)
C15	0.0254 (9)	0.0136 (10)	0.0164 (9)	−0.0001 (7)	0.0080 (8)	−0.0016 (8)
C16	0.0259 (10)	0.0196 (11)	0.0218 (10)	−0.0025 (8)	0.0125 (8)	−0.0034 (8)
C17	0.0209 (9)	0.0196 (11)	0.0247 (11)	0.0000 (7)	0.0053 (8)	−0.0068 (9)
C18	0.0253 (9)	0.0207 (11)	0.0176 (10)	0.0032 (8)	0.0056 (8)	−0.0033 (8)
C19	0.0237 (9)	0.0156 (10)	0.0138 (9)	0.0022 (7)	0.0074 (7)	−0.0013 (8)
C20	0.0279 (10)	0.0401 (14)	0.0257 (12)	−0.0037 (10)	0.0118 (9)	0.0017 (11)
C21	0.0254 (10)	0.0585 (18)	0.0252 (12)	0.0059 (11)	0.0068 (9)	0.0161 (12)
C22	0.075 (3)	0.095 (4)	0.062 (3)	0.051 (3)	0.041 (3)	0.057 (3)
C23	0.067 (3)	0.069 (3)	0.0283 (19)	−0.009 (2)	0.0221 (19)	−0.003 (2)
C22X	0.075 (3)	0.095 (4)	0.062 (3)	0.051 (3)	0.041 (3)	0.057 (3)
C23X	0.067 (3)	0.069 (3)	0.0283 (19)	−0.009 (2)	0.0221 (19)	−0.003 (2)
C24	0.0218 (10)	0.0323 (13)	0.0406 (14)	−0.0009 (9)	0.0094 (10)	−0.0063 (11)

### Geometric parameters (Å, °)

**Table:**

S1—C9	1.7327 (19)	C14—C15	1.384 (3)
S1—C10	1.816 (2)	C14—C19	1.386 (3)
O1—C6	1.379 (2)	C15—C16	1.390 (3)
O1—C7	1.417 (2)	C15—H15A	0.9300
O2—N5	1.368 (2)	C16—C17	1.396 (3)
O2—C13	1.418 (2)	C16—H16A	0.9300
O3—C13	1.204 (2)	C17—C18	1.402 (3)
N1—C8	1.307 (2)	C17—C24	1.503 (3)
N1—N2	1.405 (2)	C18—C19	1.387 (3)
N2—C9	1.309 (2)	C18—H18A	0.9300
N3—C9	1.373 (2)	C19—H19A	0.9300
N3—C8	1.373 (2)	C20—H20A	0.9600
N3—N4	1.385 (2)	C20—H20B	0.9600
N4—C11	1.293 (2)	C20—H20C	0.9600
N5—N6	1.314 (2)	C21—C23X	1.438 (11)
N6—C12	1.371 (2)	C21—C22	1.472 (5)
N6—C14	1.453 (2)	C21—C23	1.585 (5)
C1—C6	1.387 (3)	C21—C22X	1.716 (16)
C1—C2	1.397 (3)	C21—H21A	0.9800
C1—H1A	0.9300	C21—H21B	0.9600
C2—C3	1.381 (3)	C22—H22A	0.9600
C2—C20	1.511 (3)	C22—H22B	0.9600
C3—C4	1.385 (3)	C22—H22C	0.9600
C3—H3A	0.9300	C23—H23A	0.9600
C4—C5	1.390 (3)	C23—H23B	0.9600
C4—H4A	0.9300	C23—H23C	0.9600
C5—C6	1.406 (3)	C22X—H22D	0.9600
C5—C21	1.515 (3)	C22X—H22E	0.9600
C7—C8	1.494 (3)	C22X—H22F	0.9600
C7—H7A	0.9700	C23X—H23D	0.9600
C7—H7B	0.9700	C23X—H23E	0.9600
C10—C11	1.513 (3)	C23X—H23F	0.9600
C10—H10A	0.9700	C24—H24A	0.9600
C10—H10B	0.9700	C24—H24B	0.9600
C11—C12	1.449 (3)	C24—H24C	0.9600
C12—C13	1.434 (3)		
			
C9—S1—C10	93.77 (9)	C14—C15—H15A	120.7
C6—O1—C7	117.65 (16)	C16—C15—H15A	120.7
N5—O2—C13	111.22 (14)	C15—C16—C17	121.03 (19)
C8—N1—N2	107.70 (15)	C15—C16—H16A	119.5
C9—N2—N1	106.76 (15)	C17—C16—H16A	119.5
C9—N3—C8	105.11 (16)	C16—C17—C18	118.38 (18)
C9—N3—N4	129.02 (15)	C16—C17—C24	121.3 (2)
C8—N3—N4	124.59 (15)	C18—C17—C24	120.3 (2)
C11—N4—N3	115.09 (15)	C19—C18—C17	121.6 (2)
N6—N5—O2	105.30 (15)	C19—C18—H18A	119.2
N5—N6—C12	114.48 (16)	C17—C18—H18A	119.2
N5—N6—C14	113.81 (15)	C14—C19—C18	117.90 (18)
C12—N6—C14	131.68 (16)	C14—C19—H19A	121.0
C6—C1—C2	120.4 (2)	C18—C19—H19A	121.0
C6—C1—H1A	119.8	C2—C20—H20A	109.5
C2—C1—H1A	119.8	C2—C20—H20B	109.5
C3—C2—C1	118.3 (2)	H20A—C20—H20B	109.5
C3—C2—C20	121.42 (19)	C2—C20—H20C	109.5
C1—C2—C20	120.3 (2)	H20A—C20—H20C	109.5
C2—C3—C4	120.7 (2)	H20B—C20—H20C	109.5
C2—C3—H3A	119.7	C23X—C21—C5	115.5 (5)
C4—C3—H3A	119.7	C22—C21—C5	116.2 (3)
C3—C4—C5	122.6 (2)	C22—C21—C23	109.8 (4)
C3—C4—H4A	118.7	C5—C21—C23	107.9 (3)
C5—C4—H4A	118.7	C23X—C21—C22X	107.5 (8)
C4—C5—C6	115.9 (2)	C5—C21—C22X	103.8 (5)
C4—C5—C21	123.4 (2)	C22—C21—H21A	107.5
C6—C5—C21	120.61 (19)	C5—C21—H21A	107.5
O1—C6—C1	123.86 (18)	C23—C21—H21A	107.5
O1—C6—C5	114.11 (18)	C23X—C21—H21B	109.9
C1—C6—C5	122.03 (18)	C22—C21—H21B	131.2
O1—C7—C8	105.79 (16)	C5—C21—H21B	109.9
O1—C7—H7A	110.6	C23—C21—H21B	68.1
C8—C7—H7A	110.6	C22X—C21—H21B	109.9
O1—C7—H7B	110.6	C21—C22—H22A	109.5
C8—C7—H7B	110.6	C21—C22—H22B	109.5
H7A—C7—H7B	108.7	C21—C22—H22C	109.5
N1—C8—N3	109.95 (16)	C21—C23—H23A	109.5
N1—C8—C7	127.09 (17)	H21B—C23—H23A	94.7
N3—C8—C7	122.97 (17)	C21—C23—H23B	109.5
N2—C9—N3	110.46 (16)	H21B—C23—H23B	144.6
N2—C9—S1	129.40 (15)	C21—C23—H23C	109.5
N3—C9—S1	120.04 (14)	H21B—C23—H23C	85.0
C11—C10—S1	111.30 (13)	C21—C22X—H22D	109.5
C11—C10—H10A	109.4	C21—C22X—H22E	109.5
S1—C10—H10A	109.4	H22D—C22X—H22E	109.5
C11—C10—H10B	109.4	C21—C22X—H22F	109.5
S1—C10—H10B	109.4	H22D—C22X—H22F	109.5
H10A—C10—H10B	108.0	H22E—C22X—H22F	109.5
N4—C11—C12	119.20 (17)	C21—C23X—H23D	109.5
N4—C11—C10	123.66 (18)	C21—C23X—H23E	109.5
C12—C11—C10	117.04 (16)	H23D—C23X—H23E	109.5
N6—C12—C13	105.21 (16)	C21—C23X—H23F	109.5
N6—C12—C11	128.48 (17)	H23D—C23X—H23F	109.5
C13—C12—C11	126.00 (17)	H23E—C23X—H23F	109.5
O3—C13—O2	120.25 (17)	C17—C24—H24A	109.5
O3—C13—C12	135.99 (19)	C17—C24—H24B	109.5
O2—C13—C12	103.76 (16)	H24A—C24—H24B	109.5
C15—C14—C19	122.49 (17)	C17—C24—H24C	109.5
C15—C14—N6	118.37 (17)	H24A—C24—H24C	109.5
C19—C14—N6	118.95 (17)	H24B—C24—H24C	109.5
C14—C15—C16	118.53 (19)		
			
C8—N1—N2—C9	0.3 (2)	N3—N4—C11—C10	−4.3 (3)
C9—N3—N4—C11	−27.6 (3)	S1—C10—C11—N4	47.2 (3)
C8—N3—N4—C11	167.24 (18)	S1—C10—C11—C12	−136.52 (16)
C13—O2—N5—N6	1.3 (2)	N5—N6—C12—C13	−0.9 (2)
O2—N5—N6—C12	−0.2 (2)	C14—N6—C12—C13	176.92 (19)
O2—N5—N6—C14	−178.43 (15)	N5—N6—C12—C11	172.90 (19)
C6—C1—C2—C3	−0.2 (3)	C14—N6—C12—C11	−9.3 (3)
C6—C1—C2—C20	−179.7 (2)	N4—C11—C12—N6	5.8 (3)
C1—C2—C3—C4	−0.2 (4)	C10—C11—C12—N6	−170.68 (19)
C20—C2—C3—C4	179.3 (2)	N4—C11—C12—C13	178.4 (2)
C2—C3—C4—C5	0.6 (4)	C10—C11—C12—C13	1.9 (3)
C3—C4—C5—C6	−0.5 (4)	N5—O2—C13—O3	178.29 (18)
C3—C4—C5—C21	176.9 (3)	N5—O2—C13—C12	−1.8 (2)
C7—O1—C6—C1	−5.2 (3)	N6—C12—C13—O3	−178.5 (2)
C7—O1—C6—C5	174.8 (2)	C11—C12—C13—O3	7.5 (4)
C2—C1—C6—O1	−179.7 (2)	N6—C12—C13—O2	1.5 (2)
C2—C1—C6—C5	0.3 (3)	C11—C12—C13—O2	−172.44 (19)
C4—C5—C6—O1	−180.0 (2)	N5—N6—C14—C15	−48.2 (2)
C21—C5—C6—O1	2.5 (3)	C12—N6—C14—C15	133.9 (2)
C4—C5—C6—C1	0.1 (3)	N5—N6—C14—C19	126.9 (2)
C21—C5—C6—C1	−177.4 (2)	C12—N6—C14—C19	−50.9 (3)
C6—O1—C7—C8	−177.50 (18)	C19—C14—C15—C16	2.5 (3)
N2—N1—C8—N3	−1.2 (2)	N6—C14—C15—C16	177.51 (18)
N2—N1—C8—C7	179.21 (18)	C14—C15—C16—C17	−0.8 (3)
C9—N3—C8—N1	1.5 (2)	C15—C16—C17—C18	−0.7 (3)
N4—N3—C8—N1	169.57 (17)	C15—C16—C17—C24	−178.7 (2)
C9—N3—C8—C7	−178.86 (18)	C16—C17—C18—C19	0.6 (3)
N4—N3—C8—C7	−10.8 (3)	C24—C17—C18—C19	178.7 (2)
O1—C7—C8—N1	−45.2 (3)	C15—C14—C19—C18	−2.6 (3)
O1—C7—C8—N3	135.22 (19)	N6—C14—C19—C18	−177.54 (17)
N1—N2—C9—N3	0.6 (2)	C17—C18—C19—C14	1.0 (3)
N1—N2—C9—S1	−175.78 (15)	C4—C5—C21—C23X	−43.8 (7)
C8—N3—C9—N2	−1.3 (2)	C6—C5—C21—C23X	133.5 (7)
N4—N3—C9—N2	−168.63 (17)	C4—C5—C21—C22	27.4 (5)
C8—N3—C9—S1	175.50 (14)	C6—C5—C21—C22	−155.2 (4)
N4—N3—C9—S1	8.1 (3)	C4—C5—C21—C23	−96.3 (3)
C10—S1—C9—N2	−154.3 (2)	C6—C5—C21—C23	81.0 (3)
C10—S1—C9—N3	29.67 (17)	C4—C5—C21—C22X	73.6 (6)
C9—S1—C10—C11	−51.92 (16)	C6—C5—C21—C22X	−109.1 (6)
N3—N4—C11—C12	179.41 (17)		

### Hydrogen-bond geometry (Å, °)

**Table:**

*D*—H···*A*	*D*—H	H···*A*	*D*···*A*	*D*—H···*A*
C10—H10B···O3	0.97	2.20	2.993 (3)	138
C10—H10A···N1^i^	0.97	2.56	3.393 (3)	144
C15—H15A···N2^ii^	0.93	2.49	3.377 (3)	160
C19—H19A···N2^iii^	0.93	2.47	3.397 (3)	174

Symmetry codes: (i) −*x*+2, −*y*+1, −*z*; (ii) *x*, −*y*+3/2, *z*+1/2; (iii) −*x*+2, −*y*+2, −*z*.
